# Diagnostic role of stool TB-PCR in suspected tuberculous colitis patient at Dr Cipto Mangunkusumo National Central General Hospital: a cross-sectional study

**DOI:** 10.11604/pamj.2022.42.262.22293

**Published:** 2022-08-10

**Authors:** Siskawati Suparmin, Nuri Dyah Indrasari, Kaka Renaldi, Suzanna Immanuel, Yusra Yusra, Merci Monica br Pasaribu

**Affiliations:** 1 Department of Clinical Pathology, Faculty of Medicine, Universitas Indonesia, Jakarta, Indonesia,; 2 Department of Internal Medicine, Faculty of Medicine, Universitas Indonesia, Jakarta, Indonesia

**Keywords:** Polymerase chain reaction, stool, tuberculous colitis

## Abstract

**Introduction:**

the signs and symptoms of tuberculous (TB) colitis were similar with other diseases, such as inflammatory bowel disease. Therefore, finding the diagnostic modality to help differentiate TB colitis with other diseases was a challenge. In this study we aimed to find the proportion of positive stool TB-PCR in suspected TB colitis subjects and also the diagnostic value of the stool TB-PCR if compared to colonoscopy, histopathology and clinical evaluation.

**Methods:**

a cross-sectional study was done on subjects suspected to have TB colitis who undergone colonoscopy and histopathology examination between February-April 2019. Stool samples from those subjects were collected and extracted with the QIAamp® Fast Stool DNA Mini Kit. The TB-PCR was done using artus® M. tuberculosis RG PCR kit, which targeted on 16s rRNA gene. The results of stool TB-PCR then were compared with the combination of colonoscopy, histopathology and clinical evaluation as the gold standard.

**Results:**

from sixty subjects who were recruited, there were 26/60 (43.3%) subjects with positive stool TB-PC. It was consisted of 7/8 TB colitis subjects and 19/52 non-TB colitis subjects. The diagnostic value of the stool TB-PCR was: sensitivity 87.5%, specificity 63.5%, positive predictive value 26.9% and negative predictive value 97.1%.

**Conclusion:**

stool TB-PCR has good sensitivity but low specificity for diagnosing TB colitis. Therefore, stool TB-PCR could be utilized as a screening test for TB colitis.

## Introduction

Tuberculosis (TB) still became a major health problem around the world. About 10 million cases of TB around the world were reported in 2017. Indonesia was the third country with the highest TB incidence in the world. Tuberculosis commonly known for affecting the lungs, however it can also manifest as extrapulmonary TB. In Southeast Asia, there was 15% of new and relapsing TB cases in the form of extrapulmonary TB [1]. The diagnosis of tuberculous colitis (TB colitis) was challenging because its clinical manifestation and test results can resemble other diseases, such as inflammatory bowel disease (IBD) [2]. It was important to differentiate TB colitis from IBD because their clinical course were different. The majority of IBD patients were responsive to immunotherapy, but a minority were also responsive to the anti-tuberculosis drug. It made the diagnosis to be more complicated. In the other hand, steroid therapy would also be dangerous if it was given to a TB patient. Steroid therapy may induce systemic spreading of the TB and fatal complications [3,4]. Recent studies reported that the polymerase chain reaction (PCR) may play a role in differentiating between IBD and TB colitis. Amarapurkar *et al*. (2004) reported that tissue tuberculosis-polymerase chain reaction (TB-PCR) is a useful method for differentiating TB colitis from IBD. However, the positivity rate of tissue TB-PCR was low in that study [5]. Stool as the alternative specimen has advantages over tissue TB-PCR. Stool sampling was non-invasive and less affected by sampling error than tissue sampling [6]. Therefore, this study aimed to find the proportion of stool TB-PCR results in suspected TB colitis subjects and the diagnostic value of stool TB-PCR if compared with colonoscopy, histopathology and clinical evaluation.

## Methods

**Study design:** we conducted a cross-sectional study in the Cipto Mangunkusumo Hospital, Indonesia. We searched for the medical records of patients at endoscopy center from February-March 2019 to select the potential candidates.

**Population under study:** the inclusion criteria were adults (>18 years old), suspected of having clinical TB colitis, had undergone colonoscopy, were not using anti-tuberculous drugs, agreed to participate in this study and signed informed consent. The subjects were selected by consecutive sampling.

**Data collection:** data for each patient´s age, gender, clinical symptoms, clinical diagnosis, TB status, were collected from medical records. Subjects who fulfil the inclusion criteria were requested to collect their stools in a clean container. Stool samples were transported to the laboratory in less than 1-hour at room temperature or less than 6 hours at 2-8°C using a cooling bag. Stool samples then were stored at -20°C for 1-2 months until being extracted. Stool deoxyribonucleic acid (DNA) extraction was done with QIAamp® Fast Stool DNA Mini Kit. Extracted samples were then stored at -20°C before being tested for nucleic acid concentration and purity. The tests for nucleic acid concentration and purity were done by using the NanoDropTM 2000/2000c Spectrophotometer at Laboratorium Terpadu, Faculty of Medicine, Universitas Indonesia. The TB-PCR was done with the artus® *M. Tuberculosis* RG PCR kit, which targets the 16s ribosomal ribonucleic acid (16s rRNA) gene using the RotorGene Q MDx real time PCR cycler. The colonoscopy, histopathology and clinical evaluation results data were collected from the medical records in April-June 2019.

The variables to be compared were stooled TB-PCR as the test variable and combination of colonoscopy, histopathology and clinical evaluation as the gold standard test. As the gold standard for the diagnosis has not been established, we considered the combination of colonoscopy, histopathology and clinical evaluation as the surrogate measures. We categorized subjects as having TB colitis if at least two of the three tests (colonoscopy, histopathology and clinical evaluation) were positive for TB colitis The results of the PCR were categorized as negative and positive. The PCR results considered to be positive, the signal appears in the fluorescence channel cycling green at a cycle threshold (Ct) = 40. Colonoscopy, histopathology and clinical evaluation results were categorized as positive and negative for TB colitis. If the results of colonoscopy were compatible with TB colitis, then the results categorized as positive for TB colitis. It was also applies for histopathology results. For clinical evaluation, subjects were categorized as positive for TB colitis if we found the TB colitis as patient´s diagnosis as the final diagnosis or the subjects were then treated with anti-tuberculous for gastrointestinal TB from the medical record. Selection bias was avoided by using consecutive sampling method. Information bias may be the weakness of this study because we got the data from medical record for patient sign, symptoms, TB status, result of colonoscopy, histopathology and clinical evaluation. Confounding factors like TB other than colitis TB at the point of patient selection was avoided by excluding patient with antituberculosis drug therapy.

**Sample size:** the number of subjects required (n) was calculated from the sample size formula for diagnostic study below [7]:


$$n=\frac{Z\alpha^{2}\;sen(1-sen)}{d^{2}P}$$

We got data from previous study, the prevalence of TB colitis (P) was 0.27. We expected the sensitivity (sen) of stool TB-PCR to be 90% and Zα=1.96. We estimated the precision of test (d) at 15%. Then we got 57 as the minimal sample size.

**Data analysis:** the subjects with incomplete data or information needed in this study will be excluded from the analysis. The collected data were analysed with the Medcalc free statistical calculator, Statistical Product and Service Solution (SPSS) version 20 and Microsoft Excel for Office 365. Diagnostic test analyses were done using a 2 x 2 table.

The study has been approved by the Faculty of Medicine, Universitas Indonesia Ethical Committee, with licence number 1349/UN2.F1/ETIK/2018. This study was also approved to be conducted in the Cipto Mangunkusumo Hospital, licence number: LB.02/22.1/0109/2019.

## Results

**Figure 1:**
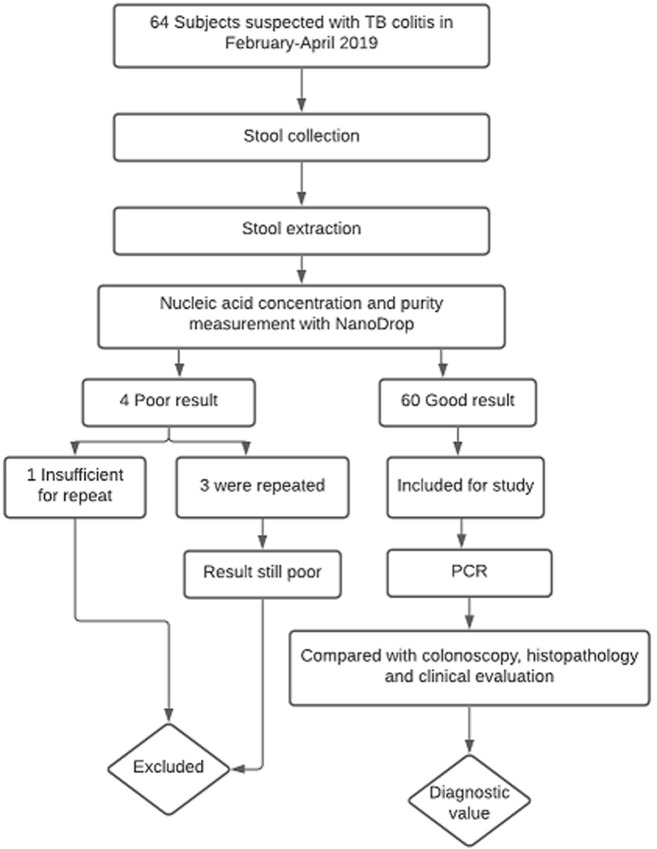
flow chart describing study participants selection and specimen’s eligibility

**Figure 2:**
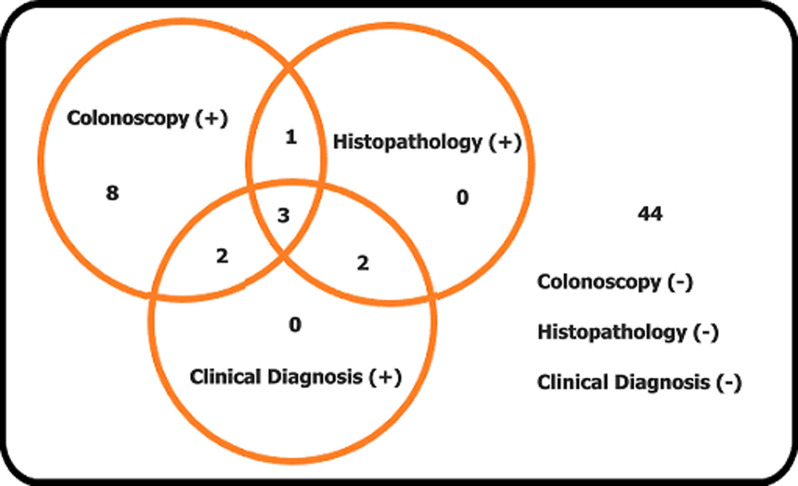
distribution of colonoscopy, histopathology and clinical evaluation results

A total of 64 subjects fulfilled the inclusion criteria. Of the original 64 subjects participating in the study, four subjects were excluded because of poor nucleic acid concentration and purity. We did re-extraction for three samples. One sample was not re-extracted because of insufficient amount of specimen. The three re-extracted sample still had poor nucleic acid concentration and purity (Figure 1). Those all three samples had a watery stool consistency. In this study, we did not do the method that concentrates the nucleic acid. Sixty subjects were therefore analysed for the diagnostic study. The subjects´ characteristic can be seen in Table 1. Only 8 from 60 subjects recruited has TB colitis. From the TB colitis group, the most common symptom was weight loss. This was different with the non-TB colitis group, in which the most common symptom was haematochezia and only few have weight loss as symptom. There were 26/60 (43.3%) positive stool TB-PCRs, consisting of 7/8 of the TB colitis subjects and 19/52 of the non-TB colitis subjects. The distribution of the colonoscopy, histopathology and clinical evaluations can be seen in Figure 2. Forty-four subjects did not meet the criteria of TB colitis from the colonoscopy, histopathology and clinical evaluation. Only three subjects fulfil all of three parameters for TB colitis (colonoscopy, histopathology and clinical evaluation). The results of the PCR, as compared to the combination of colonoscopy, histopathology and clinical evaluation as the surrogate measures of gold standard can be seen in Table 2. The diagnostic values of the stool TB-PCR were sensitivity 87.5%, specificity 63.5%, positive predictive value (PPV) 26.9%, negative predictive value (NPV) 97.1%, positive likelihood ratio (PLR) 2.39 and negative likelihood ratio (NLR) 0.20.

**Table 1:** subject’s characteristics

Characteristics	TB Colitis*	Non-TB Colitis*
	(n = 8)	(n = 52)
Age (years)	38.6 (15.76)**	49.7 (17.96)**
Gender		
Male	4 (50.0%)	23 (44.2%)
Clinical symptoms		
Abdominal pain	6 (75.0%)	24 (46.2%)
Diarrhoea	6 (75.0%)	22 (42.3%)
Constipation	0 (0.0%)	6 (11.5%)
Haematochezia	5 (62.5%)	28 (53.8%)
Weight loss	7 (87.5%)	15 (28.8%)
Adhesion/Intestinal obstruction	3 (37.5%)	0 (0.0%)
Pulmonary TB	0 (0.0%)	1 (1.9%)
Other Extrapulmonary TB	2 (25.0%)	3 (5.8%)
TB Lymphadenitis	1 (12.5%)	0 (0.0%)
TB Meningitis	0 (0.0%)	1 (1.9%)
TB Peritonitis	1 (12.5%)	0 (0.0%)
TB Pleuritis	0 (0.0%)	1 (1.9%)
TB Spondylitis	0 (0.0%)	1 (1.9%)

Note: *Grouping of TB colitis and non-TB colitis was based on operational definition of study; **mean (SD)

**Table 2:** the 2 x 2 diagnostic study table for stool TB-PCR compared to a combination of colonoscopy, histopathology and clinical evaluation

Stool TB-PCR	Combination of Colonoscopy, Histopathology and Clinical Evaluation		Total
	TB Colitis*	Non-TB Colitis	
Positive	7	19	26
Negative	1	33	34
**Total**	8	52	60

Sensitivity 87,5% (95%CI 47,35-99,68); Specificity 63,5% (95%CI 48,96-76,38); Positive Predictive Value 26,9% (95%CI 19,12-36,48); Negative Predictive Value 97,1% (95%CI 83,91-99,52) *Subjects were categorized as TB colitis if 2 of 3 variables (colonoscopy, histopathology and clinical evaluation) are positives.

## Discussion

In the TB colitis patients, the most prominent symptom was weight loss. Abdominal pain and diarrhoea were also higher in the TB colitis group than in the non-TB colitis group. This finding was similar to other studies, which stated that the most common symptoms of TB colitis are abdominal pain, weight loss and diarrhoea [4,5,8-10]. In the colitis TB group, three of the eight subjects had intestinal obstruction. Haematochezia was not significantly different between the TB colitis group and the non-TB colitis group. Lee states that extrapulmonary TB is found together with pulmonary TB in about 10-50% cases [11]. However, in this study, we found no subject in the TB colitis group who also had pulmonary TB. This finding may be as a result of the low number of TB colitis subjects in the study. The lack of the coincidence of pulmonary TB and TB colitis excludes the possibility of a positive PCR result from any source other than the digestive tract.

From the 52 non-TB colitis subjects, 19 (36.5%) had false positive stool TB-PCR result. These 19 subjects consisted of four subjects with IBD, five subjects with non-specific proctitis, colitis and ileitis, two subjects with 6 polyps, three subjects with diverticulosis, one subject with infective colitis, one subject with irritable bowel syndrome, one subject with Behcet colitis, one subject with haemorrhoid and one subject who died before the aetiology was identified. This rate for false positive results was higher than in the study by Ramadass *et al*. (2010) in India. Ramadass *et al*. (2010) found five TB-PCR positive results from 44 patients (11.3%) with Crohn´s disease [12]. The target for the PCR in this study was different from that of Ramadass *et al*. (2010) who were using IS6110, which is specific for *M. tuberculosis*. This study used 16s rRNA as its target to detect the *M. tuberculosis* complex, consisting of *M. tuberculosis, M. africanum, M. bovis, M. bovis* bacillus Calmette-Guerin (BCG), *M. microti*, and *M. pinnipedii* species [12,13]. The finding of *M. tuberculosis* in non-TB colitis subjects may be an epiphenomenon. The bacteria may incidentally enter the sample from food or other materials because of the endemicity of TB in the population. A latent tuberculosis infection (LTBI) in an IBD patient also can be reactivated by the use of immunosuppressive therapy [14]. This study used real time PCR, while Ramadass *et al*. (2010) used conventional PCR. Choi *et al*. (2015) report that real time PCR has a higher sensitivity than conventional PCR [15]. The artus® *M. tuberculosis* RG PCR kit with Rotor-Gene Q MDx can detect DNA of up to 0.23 copies/μL [13].

Of the eight subjects with TB colitis, there was one negative stool TB-PCR result (12.5%). The false negative rate was lower than that of Ramadass *et al*. (2010), who had five negative stool TB-PCRs from 24 (20.8%) subjects with TB colitis [12]. This difference could be caused by the different PCR method and target genes. Fei *et al*. (2014), who also used real time PCR, had five negative stool TB-PCR results from 29 subjects with TB colitis (17.2%) [6]. The sample from the TB colitis subject with a negative stool TB-PCR in this study had high nucleic acid concentration and good purity. The PCR result was also valid because a signal of internal control was detected. Therefore, the possibility that the negative result was due to an inhibitor can be excluded. This negative result may have been caused by the uneven distribution of mycobacteria in the stool sample. Another possibility was a change in the 16s rRNA gene sequence that can be associated with drug resistance [16]. The sensitivity of the stool TB-PCR was high (87.5%), but the specificity was only 63.5%. Therefore, stool TB-PCR was more useful for ruling out TB colitis than for confirming TB colitis diagnosis. The PPV was only 26.9%, but the NPV was high (97.1%) due to the low prevalence of TB colitis in this setting. The total study sample size was considered to be large enough. However, the number in the TB colitis group was lower than in other, similar studies. The PLR (2.39) showed minimal strength of stool TB-PCR in increasing the possibility that the patient had TB colitis. The NLR showed a moderate strength of stool TB-PCR in decreasing the possibility that the patient had TB colitis. The sensitivity of the stool TB-PCR in this study was slightly higher than in the study by Amarapurkar et al. (2004), which reported the sensitivity of conventional TB-PCR from tissue biopsy to be 21% [5]. Fei *et al*. (2014) report that the sensitivity of real time stool TB-PCR and tissue TB-PCR were 82.8% and 55.2%, respectively. These results showed that the stool TB-PCR is more sensitive than the tissue PCR. Fei *et al*. also stated that stool TB-PCR can detect *M. tuberculosis* DNA, which was released from any part of the gastrointestinal tract. Therefore, stool TB-PCR is less affected by sampling error than PCR of tissue biopsy [6].

The low specificity in this study may be caused by the surrogate gold standard method used here. As we have been known there is no gold standard for diagnosing tuberculous colitis. Prabhu *et al*. (2017) report that colonoscopy is sensitive but not specific for the diagnosing of TB colitis [17]. Histopathological examination also depends on the accuracy of the tissue sampling by the clinician and on the subjectivity of the pathologist´s opinion. The clinical evaluation is also subjective, and TB colitis can resemble Crohn´s disease. Seo *et al*. (2017) report an increasing misdiagnosis of TB colitis as Crohn´s disease as the prevalence of Crohn´s disease in Asia increases [18]. This study did not use the acid-fast bacilli stain or culture to confirm the diagnosis of TB colitis as the sensitivity of those methods is low, although they are the gold standard for diagnosing TB. Another possibility for explaining the low specificity is possible contamination in the pre-analytical and analytical steps. However, the possibility of contamination in this study was minimized by using clean and tightly closed containers and disposable pipette tips, tubes and tissue for each sample, by the decontamination of the laboratory and the equipment with ultraviolet and alcohol both before and after the extraction and PCR, and by using a negative control to rule out any contamination of the master mix.

Stool sampling was non-invasive, unlike colonoscopy, tissue biopsy or tissue PCR. Real time PCR, which was used in this study, was considered more sensitive and less likely to be contaminated than conventional PCR, which has been used by other studies. This study demonstrated the role of stool TB-PCR in ruling out a TB colitis as it has good sensitivity. The non-invasiveness of the test can be considered to be the advantage of using this modality for screening purpose. However, there is still a need to find a diagnostic modality that can confirm TB colitis diagnosis accurately, as this study showed low specificity for stool TB-PCR. The use of additional diagnostic modality combinations can be considered in future research to increase the sensitivity and specificity of test for TB colitis diagnosis as there is currently no single gold standard test for diagnosing TB colitis.

## Conclusion

Stool TB-PCR shows good sensitivity but low specificity for diagnosing TB colitis. Therefore, stool TB-PCR is better used as a TB colitis screening test.

### What is known about this topic

- *There is difficulty in diagnosing TB colitis;*
- *The use of PCR from tissue biopsy has good specificity but low sensitivity for diagnosing TB colitis;*
- *Using the stool PCR is less affected by sampling error and has higher sensitivity than tissue PCR*.

### What this study adds

- *The high sensitivity of using 16s rRNA target gene and real time TB-PCR from a stool specimen;*
- *The possibility of using stool real time TB-PCR as a screening method for ruling out a diagnosis of TB colitis*.
